# Deep psychophysiological phenotyping of adolescents and adults with 22q11.2 deletion syndrome: a multilevel approach to defining core disease processes

**DOI:** 10.1186/s12888-023-04888-5

**Published:** 2023-06-13

**Authors:** David A. Parker, Joseph F. Cubells, Sid L. Imes, Gabrielle A. Ruban, Brett T. Henshey, Nicholas M. Massa, Elaine F. Walker, Erica J. Duncan, Opal Y. Ousley

**Affiliations:** 1 Department of Human Genetics, Emory University School of Medicine, Whitehead Biomedical Research Building 615 Michael Street Suite 301, Atlanta, GA 30322 USA; 2 Department of Human Genetics; Emory Autism Center; Department of Psychiatry and Behavioral Sciences, Emory University School of Medicine, 1551 Shoup Court, Decatur, GA 30033 USA; 3 Emory University, Whitehead Biomedical Research Building 615 Michael Street Suite 301, Atlanta, GA 30322 USA; 4 Atlanta Veterans Administration Health Care System, 1670 Clairmont Road, Decatur, GA 30033 USA; 5 Department of Psychology, Emory University, Psychology and Interdisciplinary Sciences Building Suite 487, 36 Eagle Row, Atlanta, GA 30322 USA; 6 Department of Psychiatry and Behavioral Sciences, Emory University School of Medicine, Brain Health Center, 12 Executive Park Dr, Atlanta, GA 30329 USA; 7 Department of Psychiatry and Behavioral Sciences, Emory University School of Medicine, 1551 Shoup Court, Decatur, GA USA

**Keywords:** (up to 10): 22q11.2DS, DiGeorge syndrome, Velocardiofacial syndrome, Deep phenotyping, Acoustic startle, EEG, Mismatch negativity, Cognition, Psychosis risk

## Abstract

**Background:**

22q11.2 deletion syndrome (22q11.2DS) is the most common chromosomal interstitial-deletion disorder, occurring in approximately 1 in 2000 to 6000 live births. Affected individuals exhibit variable clinical phenotypes that can include velopharyngeal anomalies, heart defects, T-cell-related immune deficits, dysmorphic facial features, neurodevelopmental disorders, including autism, early cognitive decline, schizophrenia, and other psychiatric disorders. Developing comprehensive treatments for 22q11.2DS requires an understanding of both the psychophysiological and neural mechanisms driving clinical outcomes. Our project probes the core psychophysiological abnormalities of 22q11.2DS in parallel with molecular studies of stem cell-derived neurons to unravel the basic mechanisms and pathophysiology of 22q11.2-related psychiatric disorders, with a primary focus on psychotic disorders. Our study is guided by the central hypothesis that abnormal neural processing associates with psychophysiological processing and underlies clinical diagnosis and symptomatology. Here, we present the scientific background and justification for our study, sharing details of our study design and human data collection protocol.

**Methods:**

Our study is recruiting individuals with 22q11.2DS and healthy comparison subjects between the ages of 16 and 60 years. We are employing an extensive psychophysiological assessment battery (e.g., EEG, evoked potential measures, and acoustic startle) to assess fundamental sensory detection, attention, and reactivity. To complement these unbiased measures of cognitive processing, we will develop stem-cell derived neurons and examine neuronal phenotypes relevant to neurotransmission. Clinical characterization of our 22q11.2DS and control participants relies on diagnostic and research domain criteria assessments, including standard Axis-I diagnostic and neurocognitive measures, following from the Measurement and Treatment Research to Improve Cognition in Schizophrenia (MATRICS) and the North American Prodrome Longitudinal Study (NAPLS) batteries. We are also collecting measures of autism spectrum (ASD) and attention deficit/hyperactivity disorder (ADHD)-related symptoms.

**Discussion:**

Studying 22q11.2DS in adolescence and adulthood via deep phenotyping across multiple clinical and biological domains may significantly increase our knowledge of its core disease processes. Our manuscript describes our ongoing study’s protocol in detail. These paradigms could be adapted by clinical researchers studying 22q11.2DS, other CNV/single gene disorders, or idiopathic psychiatric syndromes, as well as by basic researchers who plan to incorporate biobehavioral outcome measures into their studies of 22q11.2DS.

## Background

22q11.2 deletion syndrome (22q11.2DS) is the most common chromosomal interstitial-deletion disorder known in humans, occurring in approximately 1 in 2000–6000 live births [1–3]. The syndrome typically results from microdeletions of 1.5-3 megabases (Mb) of DNA on the proximal q arm of chromosome 22. Deletions occur commonly in the 22q11.2 region due to the presence of four low-copy-repeat (LCRs) spread across the region, which then create the potential for misalignment of sister chromatids during meiosis, and subsequent formation of deletions (and duplications). The varying sizes of deletions arise from misalignment mediated by pairing of different combinations of the LCRs. The largest deletions, approximately 3 Mb in size, are located between the outermost LCRs [4, 5].

Individuals with 22q11.2DS exhibit variable clinical phenotypes that can include velopharyngeal anomalies, heart defects, T-cell-related immune deficits, dysmorphic facial features, neurodevelopmental disorders, cognitive declines in early adulthood, and psychiatric disorders ( [6, 7]. Further, most individuals who do not meet strict diagnostic criteria for any psychiatric disorder show sub-threshold symptoms of one or more disorders. The most frequently recognized psychiatric disorder is schizophrenia (SCZ), which develops in 20 to 30% of individuals by early adulthood, although autism spectrum disorder (ASD; 17 to 50% [8–10]) and attention deficit/hyperactivity disorder (ADHD; ~ 40%) also occur frequently [11–13]. Thus, 22q11.2DS is among the most robust genetic predictors of common psychiatric disorders, although the neurobiological mechanisms driving these associations are largely unknown. Studying the core cognitive, physiological, and molecular consequences of the 22q11.2 deletion and how these lead to neuronal dysfunction and clinical symptoms will allow us to unravel the basic mechanisms and pathophysiology that increase the risk of SCZ and other psychiatric disorders [14, 15]. The Emory 22q11.2DS project strongly emphasizes psychophysiological measurements of sensory detection, attention, and reactivity. Psychophysiological assessments provide unbiased, precise measures of cognition and may increase diagnostic, predictive, and outcome measurement [16–19].

The Emory 22q11.2DS psychophysiological battery comprises tasks that associate with well-known biological markers of psychiatric disorders or clinical high risk (CHR) for psychosis. With simple auditory and visual stimuli, it is possible to identify key neural deviations at the earliest stages of sensory processing [20–22], which have been shown to be deviant in psychiatric disorders [17, 18, 23–31]. Further, many of the tasks are directly translatable to animal models of 22q11.2DS, are easily implementable in a clinical setting, and could be used in future clinical treatment studies as biological targets [32–36].

A primary psychophysiological assessment for this study measures the auditory startle response and its associated latency. The *acoustic startle response (ASR)* is a reflex contraction of the skeletal musculature in response to a strong acoustic stimulus. The ASR has traditionally been used to study “pre-pulse inhibition” (PPI), although recent work has shown that response latency is more robustly related to psychiatric outcomes [37]. *Latency of the ASR (LAT)* is the time required for the startling stimulus to travel through a 3-synapse subcortical circuit that mediates the ASR and provides a putative index of general neuronal processing speed. To date, no studies to our knowledge have examined latency of ASR in 22q11.2DS in adolescents or adults. Our study focuses on latency of the response since it is linked to psychosis status and psychosis risk, is heritable in first-degree relatives (68–90%), is relatively unaffected by medication status, and is associated with cognition in prodromal schizophrenia [37, 38].

This study also relies on electroencephalography (EEG) assessments, which have been extensively used in psychiatric research and provide a non-invasive measurement of brain dynamics at a high temporal scale (millisecond). EEG signals arise from the summation of extracellular post-synaptic potentials primarily from excitatory pyramidal neurons [39, 40]. We are examining traditional event-related potentials (derived by averaging over each trial-type in voltage units) as well as stimulus-related and ongoing neuro-oscillatory activity in the time–frequency domain. This comprehensive approach has been shown to provide greater specificity of biomarkers related to psychiatric disorders, is more clearly linked to cross-species neural responses, and has also been mapped on to specific local circuit mechanisms related to inhibitory and excitatory imbalances thought to be related to psychiatric etiology.

Our EEG measures include *mismatch negativity (MMN),* the *auditory steady-state response (aSSR),* responses to the *visual oddball (OB) task,* and *resting state EEG (RS-EEG).* MMN is an evoked potential in response to unusual or “oddball” acoustic stimuli embedded within a train of repetitive acoustic stimuli. The deviant or oddball stimuli can differ from the nondeviant stimuli in duration, pitch, or both; all these trial types can be incorporated into a single paradigm. MMN is conceptually linked to novelty detection and is highly dependent on NMDA signaling [41, 42]. The impaired generation of an enhanced response to the oddball stimuli is the well-replicated MMN abnormality seen in SCZ [31, 43]. The aSSR indexes multiple aspects of auditory sensory processing, is an EEG oscillatory event arising from neuronal activity entrained to the frequency of a repetitive auditory stimulus, and is a candidate translational biomarker for psychosis [29, 44]. The visual OB task examines the evoked response to target visual cues imbedded within a train of repetitive visual stimuli, as well as indexes the neural responses related to early visual processing and its relationship to identifying target cues. Finally, RS-EEG refers to EEG recordings during a rest state, which can provide significant insight into the ongoing neural oscillations of the brain. Differences in ongoing neural oscillations have been extensively documented in SCZ and other psychotic syndromes, and these differences are related to genes associated with neural genesis and synaptic integrity [45–48]. This would provide insight in 22q11.2DS into excitatory-inhibitory imbalances observed in schizophrenia and autism spectrum disorders [49–51].

To complement the psychophysiological battery, we are capturing the range of psychiatric, behavioral, and neurocognitive clinical outcomes in 22q11.2DS. Our Emory 22q11.2DS clinical battery includes categorical and research domain criteria measures of schizophrenia, autism spectrum disorder, and other psychiatric illnesses, as well as standardized tests of neurocognition, with many of our measures matching the Measurement and Treatment Research to Improve Cognition in Schizophrenia (MATRICS) consensus battery [52] and the North American Prodrome Longitudinal study (NAPLS; [53, 54]).

Critically, in parallel to our human phenotyping, our group is using human induced pluripotent stem cell (hiPSC) technology to derive neural progenitor cells [55, 56]. Since patient-derived iPSCs capture risk alleles identical to those of the donor individual and provide a renewable source of disease-relevant human cell types, hiPSC technology offers an unprecedented opportunity to recapitulate both normal and pathological human development, thereby opening new avenues for disease modeling and drug development [57].

Our ultimate study goals are to characterize the psychophysiological responses in individuals with 22q11.2DS and use hiPSC-derived neurons as a model for studying 22q11.2 disease biology. We also test for the associations among psychophysiological phenotypes, neurocognitive performance/measures, and clinical outcome measures. Our specific study aims include 1) examining psychophysiological responses in individuals with 22q11.2DS in comparison to individuals without a serious psychiatric diagnosis, 2) identifying associations between neurocognitive and clinical measurements and psychophysiological responses, and 3) identifying the association between neuronal phenotypes and clinical and psychophysiological phenotypes. In this paper, we outline our protocols and analysis plans for our psychophysiological, neurocognitive, and behavioral data, with our hiPSC results forthcoming.

## Methods

The Emory University Institutional Review Board and the Research and Development Committee of the Atlanta Veterans Affairs Medical Center approved all protocols and consent forms. All methods have been approved in accordance with all relevant guidelines and regulations. Consent will be obtained from all subjects (or their legal guardians) prior to being enrolled in the study.

### Subject recruitment and eligibility

**Fig. 1:**
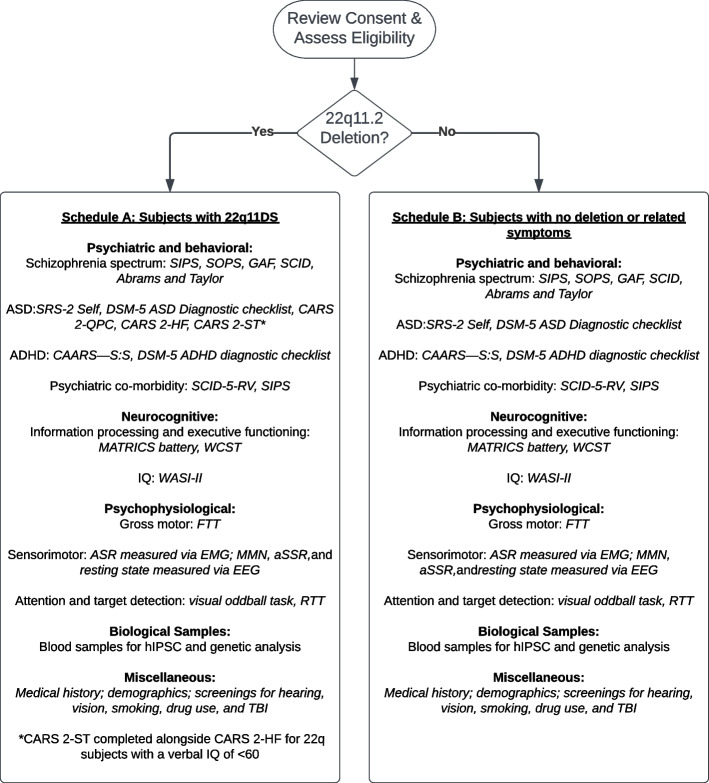
Flow chart of protocol for 22q11.2DS and healthy comparison subjects

We are acquiring data from individuals with 22q11.2DS and age-matched healthy comparison subjects between the ages of 16–60, prioritizing subjects on the younger end of this range due to their elevated risk of developing psychosis or prodromal syndromes. Study participants are primarily from the southeastern U.S.; however, our study includes individuals 22q11.2DS outside of this region. See Table 1 and Fig. 1 for overviews of study procedures and outcome measurements of each major phenotyping method.

**Table 1:** Study procedures and outcome measurements

Phenotyping measures	Primary outcome measures	Area assessed
Physiological		
Acoustic startle response	Amplitude; latency; pre-pulse inhibition	Sensory motor gating
Auditory mismatch negativity (MMN) -Frequency -Duration -Double (frequency and duration)	MMN, N100, Single-trial phase and power 4–30 HZ	N100: Basic auditory neural response MMN: Novelty detection Frequency: Low frequency oscillatory activity
Visual oddball	P100, P3a, P3b, latency	P100: Basic visual response P3a: Novelty detection P3b: Target detection and working memory
Auditory steady-state response (aSSR) -20 Hz -40 Hz -80 Hz -Chirp	Entrained oscillatory responses at the steady-state driving frequencies	Inhibitory-excitatory balance; ability to sustain entrained neural activity
5-min Resting state	Intrinsic neural oscillations across frequencies	Non-stimulus driven neural activity
Neurocognitive		
MATRICS Battery	Norm-referenced scores (T scores)	Speed of processing Attention and vigilance Working memory Verbal learning Visual learning Reasoning and problem solving
Wisconsin Card Sorting Task (WCST) - computerized	Norm-referenced perseverant response and error scores (standard scores)	Executive function Cognitive flexibility Perseverance Attention Abstract reasoning Working memory
Wechsler Abbreviated Scale of Intelligence, Second edition (WASI-II)	Norm-referenced scores (standard scores)	Verbal and nonverbal IQ
Finger tapping test	Total and average per trial tap counts (dominant and non-dominant hand)	Motor function Reaction time
Reaction time test	Latency	Motor function Reaction time
Psychiatric and behavioral		
Structured Clinical Interview for DSM-5, Research Version (SCID-5-RV)	Criteria-referenced scores (standard scores)	Presence and severity of psychiatric illness according to diagnostic criteria of the DSM-5
Structured Interview for Prodromal Symptoms (SIPS)	Criteria-referenced scores (standard scores)	Positive symptoms Negative symptoms Disorganized symptoms General psychopathology Prodromal symptoms Social, occupational, and psychological functioning Presence of psychotic symptoms Presence of lifetime brief intermittent psychotic syndrome Presence of lifetime attenuated positive symptom syndrome Presence of genetic risk and deterioration syndrome for schizophrenic spectrum disorder
Conners’ Adult ADHD Rating Scales, Self-report, Short version (CAARS:S:S)	Criteria-referenced scores (T scores)	Presence and severity of ADHD in adulthood
Social Responsiveness Scale (SRS-2)	Criteria-referenced scores (T scores)	Social deficit severity related to autism spectrum disorder
Childhood Autism Rating Scale, Second edition (CARS-2)	Criteria-referenced scores (standard scores)	Autism spectrum disorder symptoms, current and childhood presentation
Abrams and Taylor: Rating Scale for Emotional Blunting	Criteria-referenced scores (standard scores)	Affect Behavior Thought content
DSM-5 ratings	All-information-available clinical judgement	Autism spectrum disorder ADHD

### Exclusion criteria

All subjects are excluded if they have a currently unstable medical condition; a hospitalization for any medical condition within the prior 60 days; a history of neurological disease including head trauma, overt central nervous system infection, or seizure disorder not controlled by medication; a chronic autoimmune condition that increases illness susceptibility (i.e., HIV/AIDS, rheumatoid arthritis, lupus, etc.); treatment with a corticosteroid or an antibiotic in the prior 60 days; a significant hearing impairment; corrected visual acuity worse than 20/30 as indexed by eye chart screening; a full scale IQ rating of < 50; a history of illicit substance use in the prior 90 days or a positive result on urine toxicology screen; more than moderate current cannabis use; and more than moderate current alcohol use.

Healthy comparison subjects are also excluded if they had a history of psychiatric illness, current substance use disorder or neurodevelopmental disorder.

### Psychiatric and behavioral phenotyping

A primary focus of the psychiatric and behavioral assessment is identifying symptoms associated with schizophrenia and schizophrenia risk. The schizophrenia spectrum battery relies on interview measures, including the Structured Clinical Interview for DSM-5, Research Version (SCID-5-RV; [58]) and the Structured Interview for Psychosis-risk Syndromes (SIPS, 2014, version 5.6) [59–61]. The SCID-5-RV assesses a DSM-5 diagnosis of schizophrenia, in addition to mood disorders, anxiety, and substance use disorders. The SIPS examines prodromal symptom severity across four symptom domains (i.e., positive, negative, disorganized and general symptoms) and yields categorical risk syndromes (i.e., brief intermittent psychosis syndrome; attenuated positive symptom syndrome; genetic risk and functional decline syndrome; schizotypal personality disorder; DSM-5 attenuated psychosis syndrome) [62]. An additional Global Assessment of Functioning score is generated for current and past (i.e., one year prior) functioning. A dimensional examiner-generated rating scale of current negative symptoms (i.e., the Abrams and Taylor Rating Scale for Emotional Blunting; [63]) rounds out the schizophrenia symptom battery.

As 22q11.2DS associates with neurodevelopmental behavioral disorders, a battery of autism- and ADHD-related scales and ratings are included. Two measures generate dimensional scores for ASD symptoms: the Social Responsiveness Scale, Second edition (SRS-2; [64]) and the Childhood Autism Rating Scale, Second edition (CARS-2; [65]). The SRS-2 is a self-report questionnaire that generates T-scores of ASD symptoms based on a population-based sample, yielding a total score and five subdomain scores (i.e., Social Awareness, Social Cognition, Social Communication, Social Motivation, and Restricted Interests and Repetitive Behavior); the SRS-2 is administered with both 22q11.2DS and healthy comparison participants. The CARS-2 is based on all-information-available ratings and yields a T-score based on individuals with an ASD diagnosis. To obtain continuity of measures across all subjects with 22q11.2DS, the CARS-2 High-functioning (CARS-2-HF) version is completed; however, for individuals with lower IQs both the CARS-2-HF and the CARS-2 Standard version (CARS-2-ST) are completed. To assess ADHD symptoms, a self-report questionnaire is administered (i.e., the Conners' Adult ADHD Rating Scales- Self-report, Short version; CAARS-S:S; [66]) that yields gender-normed T-scores based on a population sample. The DSM-5 ratings for ASD and ADHD generate categorial outcomes for these diagnoses. All measures are used with all participants, except for the CARS-2, which is completed for only the participants with 22q11.2DS.

### Neurocognitive phenotyping

A battery of standardized measures of cognition (i.e., intelligence, memory, processing speed, and executive function), motor speed, and visual reaction time are administered within this study.

#### Cognitive battery

The Wechsler Abbreviated Scale of Intelligence, Second edition [67] measures Verbal IQ and Full Scale-2 (two subtests) IQ. The battery also assesses executive function, working memory, attention and processing speed. Specific measures include the MATRICS Consensus Cognitive Battery [52, 68–70] and the computerized Wisconsin Card Sorting Test [71] as these cognitive functions co-vary with clinical symptomatology [72].

#### Finger tapping

To measure reaction time and fine motor function, the finger tapping test and the reaction time test are administered. The finger tapping test involves the subject using their index finger to tap the key attached to a mechanical counter as many times as possible within an interval of 10 s. Six 10-s finger tapping trials are conducted for each hand (dominant and non-dominant), where all six trials for one hand must be administered before switching over to the other hand. Each trial is interrupted by a 15-s rest period. Prior to beginning this assessment, the clinical study team must note the handedness of the subject and allow the subject to complete a 10-s practice trial for each hand. For the duration of each trial, the subject must tap with their index finger while also having all other fingers flat on the mechanical counter board as well as keeping their arm and wrist flat on the table. After each trial, the total number of finger taps is recorded so that the average tap count per trial can be calculated for each hand.

#### Basic visual reaction time

This test requires the subject to click a button as soon as possible every time a black square appears on a computer screen. Prior to running the official session, the subject undergoes a 60-s practice session.

### Psychophysiological measurements

A battery of psychophysiological measures are collected to obtain metrics of unbiased sensory detection, attention, and basic cognitive processing.

#### Acoustic startle response

Acoustic stimuli are delivered binaurally through headphones and processed as previously described [38]. The startle session begins with a 1-min acclimation period of 70 decibels (dB)(A) broadband noise, which continues as the background noise throughout the session. The pulse-alone stimuli are 116 dB(A), 40 (ms) duration white noise bursts; the prepulse stimuli are 85 dB(A) 20 ms white noise bursts presented at 30, 60, and 120 ms prior to the startle stimulus. The session begins with a block of 6 pulse alone trials, followed by 3 blocks of 12 trials each. Each of these 3 blocks has 3 pulse-alone trials, plus three trials of pulse + prepulse at each 3 prepulse intervals (30, 60, and 120 ms), presented in a pseudorandom order, inter-trial intervals of 11–45 s. Finally, 6 pulse-alone trials complete the session. Blink response amplitudes are reported in microvolts. The onset latency, reported in ms, is defined by a shift of 6 digital units from the baseline value, occurring 21 to 120 ms after the pulse alone stimulus. Peak latency, also in ms, is defined as the point of maximal amplitude occurring within 150 ms from the pulse alone stimulus. On trials where no scorable blink occurs, amplitude is recorded as zero. Subjects whose mean pulse-alone amplitude in the first block is below 20 units are classified as nonstartlers as established by [73]. We compute mean latency (separately for onset vs. peak latency) for each of the 4 trial types across the 3 blocks of the session.

#### Auditory mismatch negativity (MMN)

EEG-based evoked potentials are recorded using a Brain Vision EEG recording system (Brain Vision LLC, Morrisville, NC) with 16 electrodes following the international 10–20 EEG locations with Fcz as the reference and Fpz as the ground electrode. Midsuperior and lateral to the right eye electrodes are used to detect extra-ocular movements. The Event-Related-Potential (ERP) paradigm is presented using Presentation software (Neurobehavioral Systems), as implemented in the NAPLS study: a pseudorandom sequence of frequent (90%) standard tones (633 Hz, 50 ms duration,) and infrequent (10%) deviant tones that differ in both pitch and duration (1000 Hz, 100 ms) from the standard [24, 26, 74]. Stimuli are presented at 85 dB with a stimulus onset asynchrony of 510 ms. MMN is derived by subtracting the standard ERP from the deviant ERP. MMN latency is identified in the resulting difference wave as the most negative peak between 50-265 ms. MMN amplitude is measured as the average voltage + 25 ms around this peak.

#### Data processing

Raw EEG data, processed using Brain Vision Analyzer software (Brain Vision LLC, Morrisville, NC) are reviewed to flag artifacts, then processed through a scripted pipeline performing eye movement/blink correction, bandpass filtering (0.01–30 Hz), epoching, baseline correction, artifact rejection (+ 75 microvolts), and averaging of correct trials for each stimulus type to generate ERPs. Further low-pass filtering (12–15 Hz cut-offs) are completed prior to measurement. ERP components are quantified using automated algorithms that identify peaks within pre-specified time windows to identify their latencies, and areas (e.g., ± 25 ms) centered on these peaks are used to quantify their amplitudes.

#### Auditory steady-state response (aSSR)

EEG Data is recorded using the same methods as MMN while participants complete 4 blocks of randomly interleaved 20-, 40-, 80-Hz steady-state stimuli, and a linear chirp noise (50 of each in a block; 1.2 s ISI) trials that are presented binaurally at 76 dB SPL, yielding 50 total trials at each rate. The stimuli are amplitude modulated broadband noise of 1200 ms duration (2000 ms duration for chirp).

#### Data processing

Raw EEG data are processed using Brain Vision Analyzer software (Brain Vision LLC, Morrisville, NC) as just described. Data are prepared and analyzed using previously published criteria [29]. Single-trial voltage data for each subject and sensor are converted to the time–frequency domain using EEG lab’s newtimef function [75] with a 500 ms sliding window multiplied by a 500 sample Hanning window (500 ms) in 1 ms steps from -750 pre to 1950 (2700 for chirp) ms post-stimulus onset on each trial for each sensor. A Fast Fourier Transform (FFT; 1-Hz resolution) metric is calculated at each step yielding complex numbers for time–frequency points ranging from − 500 to 1700 (2500 for chirp) ms in 1 ms bins and 1- to 100-Hz for each subject and trial-type [29]. Single Trial Power values (squared absolute values of complex FFT outputs) are then converted to decibels (10*log10). The baseline is defined as the average response from -500–0 ms. Inter-trial phase coherence (ITC) is calculated by dividing the complex FFT result by its absolute value [76, 77]. The resulting values are averaged across trials within each trial type (20, 40, 80, Chirp) using peak sensors.

#### Visual oddball (OB)

During the MMN paradigm, participants are shown a series of standard stimuli (80% of trials), target stimuli (10% of trials), and novel fractal images (10% of trials) in pseudorandom order. Participants are tasked with responding to the target stimuli with a button press. Raw EEG data are processed using Brain Vision Analyzer software (Brain Vision LLC, Morrisville, NC). EEG files are reviewed to flag artifacts, then processed through a scripted pipeline performing eye movement/blink correction, bandpass filtering (0.01–30 Hz), epoching, baseline correction, artifact rejection (+ 125 microvolts), and averaging of correct trials for each stimulus type to generate ERPs. P100 peaks are derived from sensors O1 and O2, and P3a and P3b peaks are captured via sensor PZ. ERP components are quantified using automated algorithms that identify peaks within pre-specified time windows to identify their latencies, and areas (e.g., ± 25 ms) centered on these peaks are used to quantify their amplitudes. Button responses are used to calculate target detection latency and accuracy.

#### Time–frequency

Single-trial voltage data for each subject and sensor are converted to the time–frequency domain using EEGlab’s newtimef function [75] with a 500 ms sliding window multiplied by a 500 sample Hanning window (500 ms) from -750 pre to 750 ms post-stimulus onset on each trial for each sensor in 1 ms steps. A Fast Fourier Transform (FFT; 1-Hz resolution) metric is calculated at each step, yielding complex numbers for time–frequency points ranging from − 500 to 500 ms in 1 ms bins and 1–30 Hz for each subject and trial-type [29]. Single Trial Power values (squared absolute values of complex FFT outputs) are then converted to decibels (10*log10). The baseline is defined as the average response from -100 –0 ms. Inter-trial phase coherence (ITC) is calculated by dividing the complex FFT result by its absolute value.

#### Resting state EEG

Participants fixate on a central cross for 5 min. EEG files are reviewed to flag artifacts, then processed through a scripted pipeline performing eye movement/blink correction, bandpass filtering (0.05- 55 Hz), epoching, and artifact rejection (+ 125 microvolts). Data are prepared and analyzed using previously published criteria from [47]. Data are epoched into 2000 ms bins (150 epochs). Epochs having activity ± 125 mV at any sensor at any time point are excluded from any subsequent analysis. After artifact rejection, data are transformed into the time–frequency domain in EEGlab using a Fast Fourier Transformation (FFT; 1-Hz resolution, a 50% overlapping hanning tapered window, 1–55 Hz, 1000 ms steps) resulting in 4 time-bins per epoch. Power values (squared absolute values of complex FFT outputs) will then be converted to decibels (10*log10). Power values will then be averaged over all time bins. Power values will then be averaged into frequency bands known to capture the cortical relevant frequency bands resolvable with EEG (Delta: 1–4 Hz, Theta: 4–7 Hz, Alpha: 8–12 Hz, Beta: 13–30 Hz, and Gamma: 31–55 Hz). Within each frequency band, peak sensors are selected based on the grand average response across participants.

### Anticipated sample size and power analyses

Our anticipated enrollment is between 100–150 subjects with a ratio of 2:1 22q11.2DS participants to healthy comparison subjects. Using G*Power 3.1 [78], we calculated that at power value of 0.8 and alpha of 0.05, we will be able to detect f-effect sizes of 0.26-0.32 for Group X Sex ANOVAs omnibus results and Cohen’s d-effect sizes of 0.49-0.60 for follow up two-tailed t-tests for group and sex differences.

### Overview of timeline

Visits are scheduled at least a week in advance to organize study team assignments and coordinate travel arrangements. Once the potential subject has expressed interest and has been screened for eligibility, all study visit appointments are scheduled by phone call. When specific dates and times have been orally agreed upon, the study coordinators will send the subject an email with digital copies of the study visit consents as well as written instructions that detail the overview, agenda, location, parking, and activity significance associated with the visits.

#### Day 1

The first day of testing takes place at the Emory Autism Center on Emory’s Clairmont campus a few blocks away from the main campus. After a discussion about the day’s activities and information in the consent paperwork, participants give informed consent to participate in the day’s activities. We then collect information on the subject’s demographics and medical history. For 22q11.2DS participants, a caregiver is asked to complete the CARS-2 parent questionnaire. Once we have the requisite background information, we administer the SCID and SIPS, as these instruments are the most likely to rule out a healthy comparison individual as an eligible participant. Next, the Day 1 cognitive instruments, the WASI-II and MATRICS, are administered. Finally, the subject is asked to complete the SRS-2 and the CAARS—S:S questionnaires. The subject receives compensation for participation at the end of the day. Once the subject leaves, research team members score the SIPS-related instruments, the MATRICS, the WASI-II, the Abrams and Taylor, the CARS-2, and the DSM-5 diagnostic criteria for ADHD and ASD. Initial COVID-19 precautions included a temperature screening and mask check for research participants (and parent/support person, if present) before entering the building. Participants were also asked to sign a form acknowledging Emory’s COVID-19 exposure and contact tracing policies. COVID-19 procedures have varied according to Emory University policy.

For the Day 1 assessments, the research team members were trained on the SCID-5-RV, SIPS, WASI-II, and MATRICS assessments following standard lab procedures. A combination of methods are used for training, with research team members watching standard training videos (i.e., SCID-5-RV), completing in-person or Zoom training (i.e., SIPS), and completing didactic training within the lab (i.e., WASI-II, MATRICS, and rating scales). Next, each team member is supervised by a PhD-level or highly experienced team member during subject data collection. For structured interview measures, team members complete at least one live co-coding sessions (without administration) and at least two supervised administrations/co-coding sessions. For direct assessments, at least three administrations are supervised and co-coded. Consensus coding meetings, led by a PhD-level supervisor or highly experienced research team member, are completed for all assessments and rating instruments. Detailed feedback on administration procedures is also provided. To become fully trained, a research team member is required to collect the full battery of assessments on three research participants, with at least one having 22q11.2DS. For quality control, data are examined on a periodic basis to ensure that obtained scores are within the expected range.

#### Day 2

The second day of testing takes place at the Atlanta Veterans Affairs Medical Center hospital. Most of the day takes place in a research laboratory room with equipment for conducting EMG and EEG recordings. The participant (along with their support person, if present) is asked again about recent COVID-19 symptoms, and they participate in a discussion about the day’s activities as well as their rights as a research subject. They sign the VA’s informed consent paperwork and Notice of Privacy Practices. Next, the subject is screened for hearing, vision, smoking history, traumatic brain injury history, current medications, drug use, and pregnancy (if applicable). If the subject meets these inclusion criteria, we begin to collect response data. Participants complete the WCST, reaction time test, and finger tapping test exercises. To prepare subjects for EMG recording, we attach two electrodes to the orbicularis oculi under the right eye and place a ground electrode on the right mastoid. The subject is then placed in a soundproof booth to undergo the ASR protocol detailed above. When finished, we remove the EMG electrodes and place a cap with EEG electrodes on their head and an additional electrode underneath the right eye. The subject returns to the soundproof booth for the EEG protocols. After these measurements are complete, we remove all electrodes, pay the subject for their participation, and bring the subject to a medical lab for a blood draw.

For the Day 2 assessments, the research team members are trained on the administration of the WCST, finger tapping test, basic visual reaction time task, acoustic startle response, auditory mismatch negativity/visual oddball responses, auditory steady-state response, and resting state EEG assessments following the predetermined and prewritten standard operating procedures for the lab. Research team members complete didactic training within the lab. Each team member is supervised by a PhD-level or highly experienced team member during subject data collection. Detailed feedback on administration procedures are also provided. To become fully trained, a research team member is required to collect the full battery of assessments on three research participants, with at least one having 22q11.2DS. Data are examined on a periodic basis to ensure that obtained scores are within the expected range.

#### Laboratory interface

Blood samples are obtained by a phlebotomy-trained research coordinator or, in the case of difficult draws, a designated research nurse. We collect nine tubes: three gold serum tubes and six violet EDTA plasma tubes.

#### Data management

Data are entered into a secure REDCap database where they can be accessed by all pertinent study personnel. Psychophysiological data are processed in an Access database before summary variables are imported into REDCap.

#### Multiple-site collaboration

During weekly meetings, all PIs and researchers meet and discuss relevant clinical and phenotypic responses.

## Discussion

The aim of the Emory 22q11.2DS project is to use a multilevel approach to examine the disruptions of behavioral, neuronal, and genetic phenotypes that are seen in 22q11.2DS. Our study focuses on adolescents and adults who are at higher risk of developing psychosis. Due to the complex and heterogeneous developmental trajectories and outcomes of individuals with 22q11.2DS, deep phenotyping is required to understand the condition’s clinical manifestations, psychophysiological phenotype, neurocognition, and course.

Data generated from this study will allow us to identify specific psychophysiological and biological markers that are associated with risk and resilience in 22q11.2DS. The chosen dense battery of psychophysiology and neurocognitive tasks has been extensively shown to be linked to psychosis and other neuropsychiatric disorders. Each measure was carefully selected to focus on measures that have endophenotypic qualities in psychosis, have high levels of reliability, and are related to NMDA and GABAergic neurotransmitters that are long-term targets for molecular and psychopharmacologic interventions [17, 18, 28, 37, 38, 68, 79–84]. Through deep phenotyping we will be able to examine associations across multiple dimensions of neurobiology. Many of the measures we collected use identical protocols as the North American Prodrome Longitudinal Study (NAPLS); incorporating identical protocols will enable us to compare our sample of 22q11.2DS individuals who are at high genetic risk of developing a neuropsychiatric disorder to participants in NAPLS, who are at high risk for psychosis based on their behavioral phenotypes. Across behavior and psychophysiology, we will be able to contrast our findings with a heterogeneous clinical population at high risk to identify shared and unique markers associated with risk. We will also examine how continuous measures of cognition, autism traits, and ADHD behaviors vary with psychosis and psychophysiological phenotypes, yielding a complete phenotypic profile. In parallel to our human phenotyping, we are collecting biospecimens (blood) from each participant, which are stored for future planned genetic analysis; storing these blood samples will allow our group to study potential molecular mechanisms related to 22q11.2DS and subsequently compare data across behavioral and biological dimensions. Our plans include analyses of genes and gene expression, neuro-inflammatory markers, and the development of hiSPCs neurons that will allow us to directly our human psychophysiological to cell-level neural electrophysiology.

Like many studies, the COVID-19 pandemic has caused a number of delays and updates to our safety protocols to ensure that our research participants and the researchers have minimized risk. This is especially important for individuals with 22q11.2DS who are immunocompromised and at greater risk of comorbid complications or morbidity [6]. This has created challenges in recruitment and opportunities for family engagement and education and we have to continually fine-tune our strategy.

Ultimately, we hope that our study will provide a strong foundation for future studies that engage and support the 22q11.2DS community. Our paradigms and protocols capture a wide range of behaviors and biological measures that could aid in the long-term care and management of 22q11.2DS. Our protocol also provides a comprehensive template that could be applied to other rare CNV disorders that will allow for trans-diagnosis comparisons [85, 86].

## Data Availability

Materials used are referenced in the manuscript.

## Abbreviations

22q11.2DS: 22Q11.2 deletion syndrome
ADHD: Attention deficit/hyperactivity disorder
ASD: Autism spectrum disorder
ASR: Acoustic startle response
aSSR: Auditory steady-state response
CAARS-S:S: Conners’ Adult ADHD Rating Scales-Self-report, Short version
CARS-2: Childhood Autism Rating Scale, Second edition
CHR: Clinical high risk (for psychosis)
dB: Decibels
DSM-5: Diagnostic and Statistical Manual of Mental Disorders – Fifth edition
EEG: Electhoroencephalography
EMG: Electromyography
ERP: Event-related potential
FFT: Fast Fourier transform
GABA: Gamma-aminobutyric acid
HC: Healthy comparison subjects
hiPSC: Human induced pluripotent stem cell
iPSC: Induced pluripotent stem cell
LAT: Latency of the ASR
LCR: Low-copy repeats
MATRICS: Measurement and Treatment Research to Improve Cognition in Schizophrenia Consensus Cognitive Battery
Mb: Megabase
MMN: Mismatch negativity
NAPLS: North American Prodromal Longitudinal Study
NMDA: N-methyl-D-aspartate
OB: Oddball
PPI: Pre-pulse inhibition of the ASR
RS-EEG: Resting state electroencephalography
SCZ: Schizophrenia
SIPS: Structured interview for prodromal symptoms
SOPS: Scale of Prodromal Symptoms
SRS-2: Social Responsiveness Scale, Second edition
